# Increasing H3K9 Methylation Level Reduces the Proliferation of Leukemic Stem Cells

**DOI:** 10.3390/cancers18182969

**Published:** 2026-09-14

**Authors:** Barbara Walter, Polina Zjablovskaja, Sara Montserrat-Vazquez, Eva Mejia-Ramirez, Laia Solé-Castilla, Javier Lozano-Bartolome, Miroslava Kari Adamcová, Meritxell Alberich-Jorda, Chang Sun, Qin Peng, Michael Lübbert, Amanda Amoah, Liam MacPhee, Melika Bakharzi, Florian Kuchenbauer, Arefeh Rouhi, Jasson Villarreal-Hernandez, Montserrat Arnan-Sangerman, Maria Carolina Florian

**Affiliations:** 1 Stem Cell Aging Group, Program of Regenerative Medicine, The Bellvitge Institute for Biomedical Research (IDIBELL), L’Hospitalet de Llobregat, 08908 Barcelona, Spainsmontserrat@idibell.cat (S.M.-V.); emejia@idibell.cat (E.M.-R.); lsole@idibell.cat (L.S.-C.); jlozano@idibell.cat (J.L.-B.); 2 Program for Advancing the Clinical Translation of Regenerative Medicine of Catalonia (P-CMR[C]), 08908 Barcelona, Spain; 3 Institute of Molecular Medicine, University of Ulm, 89069 Ulm, Germany; 4 Center for Networked Biomedical Research on Bioengineering, Biomaterials and Nanomedicine (CIBER-BBN), 28029 Madrid, Spain; 5 Laboratory of Haemato-Oncology, Institute of Molecular Genetics of the Czech Academy of Sciences (IMG), 142 20 Prague, Czech Republic; miroslava.adamcova@img.cas.cz (M.K.A.); meritxell.alberich-jorda@img.cas.cz (M.A.-J.); 6 Shenzhen Bay Laboratory (SZBL), Institute of Systems and Physical Biology, Shenzhen 518132, China; sunchang@szbl.ac.cn (C.S.); pengqin@szbl.ac.cn (Q.P.); 7 Department of Hematology, Oncology, and Stem Cell Transplantation, Faculty of Medicine and Medical Center, University of Freiburg, 79110 Freiburg, Germany; michael.luebbert@uniklinik-freiburg.de; 8 Terry Fox Laboratory, BC Cancer Research Institute, Vancouver, BC V5Z 1L3, Canada; aamoah@bccrc.ca (A.A.); fkuchenbauer@bccrc.ca (F.K.); arouhi@bccrc.ca (A.R.); 9 Leukaemia/Bone Marrow Transplant Program of British Columbia, Vancouver General Hospital, BC Cancer, Vancouver, BC V5Z 1M9, Canada; 10 Department of Medicine, University of British Columbia, Vancouver, BC V6T 1Z3, Canada; 11 Institut Català d’Oncologia, The Bellvitge Institute for Biomedical Research (IDIBELL), L’Hospitalet de Llobregat, 08908 Barcelona, Spain; jvillarreal@iconcologia.net (J.V.-H.); marnan@iconcologia.net (M.A.-S.); 12 The Catalan Institution for Research and Advanced Studies (ICREA), Passeig Lluís Companys, 23, 08010 Barcelona, Spain

**Keywords:** HoxA9-Meis1, acute myeloid leukemia (AML), aging, hematopoietic stem cell (HSC), histone 3 lysine 9 methylation (H3K9 methylation), IOX1

## Abstract

**Background/Objectives**: Genetic and epigenetic alterations accumulate throughout life in hematopoietic stem and progenitor cells (HSPCs), leading to an increased risk of age-related hematological malignancies, such as acute myeloid leukemia (AML). While a few epigenetic drugs have entered clinical practice, studies focusing on histone post-translational modifications (PTMs) altered in AML remain limited. **Methods**: Here, we used murine HSPCs, murine AML mouse models, human leukemic cell lines, and leukemic patient samples to investigate whether targeting the methylation of histone 3 at lysine 9 (H3K9 methylation) affects HSPCs upon aging and leukemogenesis and might represent a possible target. **Results**: Our data show that H3K9 methylation changes upon aging in HSPCs and is linked to a pre-malignant phenotype. Low levels of H3K9 methylation in leukemic cells are required to maintain proliferative capacities in vitro. Increasing H3K9 methylation reduces the leukemogenesis of both young and aged murine and patient-derived leukemic cells. **Conclusions**: Thus, H3K9 methylation might be a potential and selective therapeutic target in AML patients.

## Simple Summary

Epigenetic alterations accumulate throughout life in hematopoietic stem and progenitor cells (HSPCs), leading to an increased risk of acute myeloid leukaemia (AML). Here, we investigated whether targeting the methylation of histone 3 at lysine 9 (H3K9 methylation) affects HSPCs upon aging and leukemogenesis and might represent a possible target. Our data support the idea that low levels of H3K9 methylation in leukemic cells are required to maintain proliferative capacities in vitro. Increasing H3K9me2 reduces the leukemogenesis of both young and aged murine and patient-derived leukemic cells. Thus, H3K9me2 might be a potential and selective therapeutic target in AML patients.

## 1. Introduction

Hematopoiesis is a tightly regulated, hierarchical process in which hematopoietic stem cells (HSCs) at the apex generate all-mature blood cells and platelets [1,2]. As an organism ages, HSCs and myeloid cells expand while lymphoid differentiation capacity declines. Aging is also characterized by the gradual accumulation of genetic and epigenetic alterations, which are associated with an increased incidence of hematologic malignancies, particularly acute myeloid leukemia (AML) [3,4]. 

AML is an aggressive, age-related heterogeneous hematopoietic disease. According to the Global Cancer Observatory (GLOBOCAN), the mean incidence of AML in Europe is 5.06 per 100,000 people, with an exponential increase in incidence rate after 65 years of age [5]. AML is driven by distinct mutations in HSPCs. These mutations disrupt genes involved in HSC self-renewal and differentiation, such as *HOX* family genes, causing the expansion of mutated clones in the bone marrow (BM) [6]. Some of these mutations occur in genes that encode epigenetic regulators crucial for histone PTMs, which are often associated with both AML and aging, ultimately resulting in a proliferative advantage of transformed cells [7].

Interestingly, previous studies demonstrated that histone H3 lysine 9 methylation (H3K9 methylation) is altered in HSPCs upon aging and cancer [7,8,9,10]. Typically, H3K9 occurs as dimethylated (me2) or trimethylated (me3) and is important for maintaining the structure of heterochromatin, including transposable elements and satellite repeats, where it ensures transcriptional silencing, overall regulating gene expression, cell differentiation, self-renewal, and apoptosis mainly by downregulating gene expression [11,12,13,14,15]. In AML, H3K9 methylation patterns can shift, leading, for example, to the abnormal accumulation of H3K9 methylation at promoter regions of tumor suppressor genes [6]. This can support leukemic transformation by promoting unchecked proliferation and preventing normal differentiation [7].

Currently, the most common AML treatment strategy targets blast cell proliferation with a cytarabine/anthracycline-based regimen. These treatments are highly toxic to both healthy and malignant bone marrow cells, and recently there has been increasing interest in targeting epigenetic alterations that can induce differentiation or apoptosis in AML blasts, offering a more targeted and potentially less toxic approach. However, the role of H3K9 methylation in leukemogenesis and the self-renewal of leukemic cells and whether it could represent a potential therapeutic target is not yet completely understood [16,17,18].

Here, we investigate the role of H3K9 methylation in aging HSPCs and whether it is affected by hematopoietic differentiation and/or aging contributing to leukemogenesis and disease aggressiveness. We establish young and old myeloid leukemia mouse models based on *HoxA9*/*Meis1* overexpression to investigate how changes in H3K9 methylation could influence young and aged murine leukemic stem cell (LSC) self-renewal by targeting H3K9 methylation either pharmacologically or genetically. Our results show that, independently from the age of the leukemic cells, targeting H3K9 methylation in murine LSCs, in human AML cell lines, and in patient-derived primary LSCs decreases leukemogenesis.

## 2. Materials and Methods

### 2.1. Reagents

A list of reagents, chemicals, commercial kits, and antibodies is provided as a Source Data file.

### 2.2. Cell Lines

PhoenixEco (CRL-3214), NIH-3T3 (ACC 59), SKM-1 (ACC 547), UCSD-AML1 (ACC 691) OCI-AML3, and SKNO-1 (ACC 690) cell lines were used in this study following DSMZ and ATCC culturing directions.

### 2.3. Mice

Young and aged C57Bl/6 mice were obtained from the internal divisional stock (originally purchased from The Jackson Laboratory, Bar Harbor, Maine, USA). The NBSGW mice (JAXStock No.026622) were maintained as homozygotes. All mice were housed in the animal barrier facility under pathogen-free conditions at the Biomedical Research Institute of Bellvitge (IDIBELL). Young mice were aged between 10 and 20 weeks old and aged mice were at least 80 weeks old. All mice were randomized for sex. For the transplantation study, NBSGW mice were randomized for sex and equal numbers of male and female mice were used across samples. Mice that failed to recover from blood sampling and mice that died due to laboratory errors were excluded. Mice that needed to be euthanized because they were scored as “weak and about to die” according to our approved animal license protocol for evaluating mouse health status remained part of the dataset. Allocation to the control or treated group was done randomly (4–5 mice in each experimental group in four different experimental batches). All animals were kept according to the recommendations of the European Convention for the Protection of Vertebrate Animals used for Experimental and other Scientific Purposes (ETS 123). Animals were housed in groups of up to 4 animals per cage in Macrolon Type II (long) cages with bedding and paper nesting material. The animals had access to food (V1124-3, ssniff^®^) and water ad libitum. Animals were kept at a day/night rhythm of 12/12 h throughout the entire experiment. 

### 2.4. FACS Staining and Sorting

Mononuclear BM cells were isolated by low-density centrifugation (Histopaque1083, Sigma) and stained with a cocktail of biotinylated lineage rat anti-mouse antibodies: CD11b (M1/70), B220 (RA3-6B2), CD5 (53-7.3), Gr-1 (RB6-8C5), Ter119 (Ter119), and CD8a (53-6.7) (eBioscience). After lineage depletion by magnetic separation (Dynabeads, Invitrogen), cells were stained with Sca-1 (D7, eBioscience), cKit (ACK2), Streptavidin (eBioscience), and SytoxBlue (Invitrogen). FACS data are depicted as indicated in the figures. FACS data are plotted as myeloid progenitor (MP, Lin-cKit+Sca1-), HSPC (LSK), HoxA9/Meis1 leukemic cells (HM, Lin-cKit+GFP+), and H3R9 or H3K9 (MP or LSK+mCherry+).

### 2.5. Retroviral Vector Construction and Viral Production

Murine stem cell virus (MSCV)-based retroviral vectors carrying expression cassettes consisting of *Hoxa9* and *Meis1* have been described previously [19]. 

H3K9 and the mutant-form H3R9 sequences were kindly provided by the laboratory of Prof. Clemens Schmitt [20]. They are based on the H3.1 histone isoform with the K9 mutated to R9. Sequences were subcloned into the retroviral backbone pMSCVII (pMSCVII was a gift from Maki Nakayama (Addgene plasmid # 162750; http://n2t.net/addgene:162750, accessed on 10 February 2022; RRID:Addgene_162750) adding by PCR a P2A site and mCherry as a reporter. Resultant vectors were transfected into Phoenix-ECO using Lipofectamine 2000 (Thermo-Fisher) following the manufacturer’s instructions. Supernatant containing retroviral particles was collected at 48 h and 72 h post-transfection and kept at 4 °C until final concentration was achieved 72 h post-transfection. We used Millipore Centricon Plus-70, Ultracel-PL Membrane 100kDa (UFC710008 Millipore) to measure viral concentration. Concentrated retroviral particles were kept at −80 °C prior to transduction. Retroviral particles were titrated in dilutions ranging from 1/10 to 1/500,000 in NIH-3T3 cells (mouse embryonic fibroblasts). The titration was analyzed 48 h later by assessing mCherry-frequencies by flow cytometry (Cyto Flex SRT, MoFlo Cell Sorter). Viral infectious units (VIUs) were calculated based on the initial cell input, dilution, mCherry frequency, and volume of retroviral supernatant. The resulting values were plotted, and the average, representing the transducing units (TUs), was calculated from the linear portions of the graph.

### 2.6. Immunofluorescence Staining and Analysis

FACS-sorted cells (MP, HSPC, HM, H3R9/K9) were immunostained as previously described [7]. Freshly sorted cells were seeded on fibronectin-coated glass coverslips and incubated at the 12–16 h range in HBSS (LSK) or IMDM (HM, MP) + 10% FBS + 1% P/S at 37 °C, 5% CO_2_, and 3% O_2_. After cells were fixed with BD Cytofix Fixation Buffer (BD Biosciences), they were gently washed (PBS), permeabilized with 0.2% Triton X-100 (Sigma) in PBS (20 min), and blocked with 10% Donkey serum (Sigma, 30 min) in 0.1% TritonX-100 in PBS. Antibodies against H3K9me2 (ab1220) and H3K9me3 (ab8898) were used as primary antibodies in 0.1% TritonX-100 in PBS, and incubated for 1 h at room temperature. After one washing step with 0.1% TritonX-100 in PBS for 10 min, cells on coverslips were incubated with secondary antibody in the presence of 1.5% Donkey serum in 0.1%Triton-X100 in PBS. Secondary antibody (AF488 anti-rabbit, AF488-antimouse, Cy3-antimouse from Jackson Laboratories) incubations were performed for 1 h at room temperature, then washed in 0.1% TritonX-100 PBS, stained with DAPI, and mounted after 2 washes with Prolong Gold (Invitrogen). Samples were imaged with Zeiss LSM880 or Zeiss LSM980 equipped with a 63× objective (Zeiss, Jena, Germany). Per acquisition, 15–30 cells were analyzed per sample. A 3D reconstruction was done using Volocity v9.2. Resulting 3D volumes of H3K9me2 and -me3 were normalized to DAPI volumes. 

### 2.7. Cell Transplantation in C57Bl/6, BoyJ, and NBSGW and Transduction

For H3R9 and H3K9 LSK transplantations, young C57Bl/6 were used as donors and recipients. A total of 0.2-million LSK samples were sorted into 96-well suspension plates in IMDM, 10% FBS, and 1% P/S at 100 ng/mL TPO, SCF, and mG-CSF for transduction. Transduction efficiency was assessed by mCherry expression through flow cytometry. Transduced cells were harvested, washed, and mixed with 0.3-million competitor cells in HBSS 1%P/S and transplanted into young C57Bl/6 mice (9 Gy). Successful peripheral blood chimerism was determined by a minimum of 1% mCherry expression. 

HoxA9/Meis1 plasmids were kindly provided by Dr. Arefeh Rouhi [19]. Young and aged C57Bl/6 donor BM cells were lineage depleted as described above and co-transduced with HoxA9-GFP and Meis1-YFP retroviral vectors. A bulk of 1-million transduced cells were transplanted into 11 Gy irradiated BoyJ recipient mice. Leukemia development was monitored (WBC 15–20 million cells/mL, 10–20% weight-loss, movement, pain indicators) and PB chimerism was determined by flow cytometry (GFP/YFP expression). BM of leukemic mice was harvested and stored or used for subsequent experiments and secondary transplantations. Leukemic HM cells were FACS stained and sorted into Terasaki plates as described earlier and transplanted into non-irradiated NBSGW mice. The sufficient amount of engrafting leukemic stem cells was determined by ELDA [19]. Statistical analysis was performed according to Hu and Smyth [21] and calculated with a cutoff of 50 days, LSC estimations are depicted accordingly. Here, 1, 5, 10, 50, and 100 HM cells were transplanted in NBSGW for the statistical analysis. For the characterization of young and aged HM mice, 50 HM cells were transplanted. For EdU and apoptosis in vitro assays, the manufacturer’s instructions were followed. To optimize the timing of leukemia development (3 weeks) in mice, 50 young HM cells and 250 aged HM cells were transplanted. For the characterization of young and aged HM mice, 50 HM cells were transplanted. For EdU (Invitrogen C10418) and apoptosis (BD, 559763), the manufacturer’s instructions for in vitro assays were followed. For H3R9 or H3K9 overexpression on HM leukemic cells, we thawed vials of harvested HM leukemic cells (Lin-, cKit+) from primary transplanted mice that developed the disease in vivo (3–4 months after transplant). We cultured them overnight in StemSpan^TM^ SFEM (STEMCELL Technologies #09650) with 100 ng/mL of mSCF, 500K cells per well, and 5 wells of 24 wells/plates for H3K9 and H3R9 retroviruses. Transduced HM leukemic cells (mCherry+) were sorted and used for CFU (Methocult 3234) for self-renewal assessment.

### 2.8. Flow Cytometry of Peripheral Blood (PB)

PB cell immunostaining of HM mice cells was performed according to standard procedures and samples were analyzed on a flow cytometer (Cytoflex, Gallios). For PB lineage analysis, the antibodies used were CD3 (145-2C11), Gr1 (RB6-8C5), Mac1 (M1/70), B220 (RA3-6B2), and cKit (2B8). Flow cytometric analysis is plotted as the percentage of total events. PB immunophenotyping of patient-derived xenograft mice was performed using Lineage Cocktail (Invitrogen), CD45 (HI-30, BioLegend), GPR56 (CG4.rMAb, BD Bioscience), and CD33 (WB53, BD Bioscience) in the presence of Brilliant Violet Tandem AB Brilliant Stain Buffer (BD Bioscience).

### 2.9. Processing of Patient-Derived Samples, Screening, Sorting, and Transplantation

Patient-derived BM aspirates were diluted at a 1:1 ratio with PBS/3% FBS and homogenized using an 18-gauge blunt-end needle (BD) and 2 mL syringe. The homogenized cells were filtered through a 70 μm strainer (Corning) and counted using the Haematocounter (MindRay BC-2800 Vet, Shenzhen, China). After the cells were adjusted to 20 million cells/mL and either layered on Lymphoprep (STEMCELL Techn.) following the manufacturer’s instructions to isolate mononucleated cells or diluted and subjected to red blood cell lysis (Bio Legend). Adequate cell concentrations were prepared for cryopreservation at −80 °C in FBS, 10% DMSO. Frozen BM aspirates were screened for CD34 and GPR56 expression. For this, frozen BM aspirates were kept on dry ice until they were rapidly thawed in 37 °C water baths and resuspended in at least 10 mL IMDM and 20% FBS, DNaseI (0.01 mg/mL; STEMCELL Techn.). A total of 1 million cells were blocked using human TrueStain FcBlock (Bio Legend) for 30 min at 4 °C, after they were immediately stained with the following products: Lineage (Invitrogen), GPR56 (CG4.rMAb, BD Bioscience), CD34 (581, BD Bioscience), SytoxBlue (Invitrogen), and Brilliant Violet Tandem AB Brilliant Stain Buffer (BD Bioscience)—1 h at 4 °C. For enriching human leukemic stem cells, BM aspirates were additionally subjected to dead cell removal (EasySep™ Annexin V, STEMCELL Techn.) prior to blocking. Sytox-Lin-GPR56-hLSCs were sorted into HBSS 1% P/S and stored on ice for additional experiments. A total of 0.3 million sorted hLSCs were transplanted by i.v. into the teil vein of irradiated NBSGW mice (1.6 Gy). PB chimerism was determined by flow cytometry (Cyto Flex SRT) every four weeks up to four months after transplantation. The transplantation was performed at the Terry Fox Laboratory (British Columbia Cancer Agency). Transplanted mice were regarded as engrafted with a PB chimerism of 0.5%.

### 2.10. Colony Assay HM Cells and Patient-Derived Cells

A total of 200 or 1000 HM cells were grown in Methocult M3234 (STEMCELL Techn.) + 100 ng/mL mSCF (Prospec)a and 1000 LSK in M3534 (STEMCELL Techn.); 1000 patient-derived BM cells were cultured in H4535 (STEMCELL Techn.) and IMDM and 10% FBS. Colony assay was performed according to the manufacturer’s instructions with decreased volumes; all cell concentrations were adjusted to fit a 1:10 (*v*/*v*) ratio of cell suspension:Methocult. For the IOX1 sensitivity of HM cells, Methocult contained 12.25 µM, 25 µM, and 50 µM of IOX1 (Tocris Biotechne) or DMSO. Cells were incubated at 37 °C with 5% CO_2_ at normoxic conditions for up to 2 weeks. Colony scoring followed the instructions of StemCell Technologies. HM and LSK scoring were done using the AxioObserver Z1 microscope (Zeiss) with 10× magnification. Each set of treatment was performed in duplicates and independently repeated at least 3 times. IOX1 data are averaged and normalized to DMSO. Patient-derived colonies were scored using 20× magnification. Each duplicate was averaged, and IOX1 averages were normalized to DMSO averages. For the self-renewal capacity assessment of HM leukemic cells overexpressing H3K9 and H3R9, plating was done using 200 cells first, and colonies were scored after 2 weeks. Then, Methocult was dissolved in IMDM, 10% FBS, and 1% P/S; cells were counted; and 1000 cells were used per well in the second plating. After 2 weeks, colonies were scored again, and we repeated the process for the third plating, scoring the colonies after 2 weeks post-plating.

### 2.11. Cell Culture and OCI-AML3 Western Blot

All cell lines were cultured according to standard procedures. OCI-AML3 (purchased by ATCC) was cultured in IMDM, 10% FBS, and 1% P/S supplemented with a 50–200 µM range of IOX1 in DMSO (Tocris Biotechne) and harvested after 3, 7, 14, 20, and 40 h. The medium was changed every 24 h. Harvested cells were washed twice with cold PBS and resuspended in cold PBS and protease inhibitor (Roche cOmplete). Cells were processed with NE-PERTM (Thermo Fischer) to extract the protein nuclear fraction for histone analysis in WB. The nuclear extract of a minimum of 1M cells was used loaded on a 12% SDS-PAGE (TGXTM Miniprotean, BioRad). The protein concentration of cell lysates was determined by standard BSA assay procedures (Pierce™ BCA Protein Assay Kit). Proteins were separated using precast gradient gels (Invitrogen), transferred on nitrocellulose membranes, washed in TBS-T (0.1% Tween20), and blocked in TBS-T 4% BSA (1 h, room temperature). Anti-H3K9me2 (1:1000), anti-H3K9me3 (1:1000), and anti-H3 (1:1000) primary antibodies (all Invitrogen) were incubated overnight at 4 °C and detected with HRP-goat anti-rabbit antibody (Biorad 1/10,000) was diluted in 2% BSA in TBS-T for 2 h at a range 20–25 °C. Membranes were developed by UV reveal in BioRad’s ChemiDoc and analyzed using Image Lab 6.1 software (BioRad). The membranes were stripped by boiling them in water for 15 min. Depicted data are normalized to background, total protein load, and DMSO. 

### 2.12. Bulk RNA Sequencing and Analysis

Bulk RNA-seq libraries were generated from 2000 young LSK samples transduced with H3R9/K9 or young and aged HoxA9/Meis1 (HM) cells. The cells were cultured overnight at 37 °C, 5% CO_2_, normoxia in IMDM, 10% FBS, and 1% P/S (100 U/mL), and HM cells were treated with 50 μM of IOX1 (Tocris Biotechne #4464) or DMSO. Before processing, the cells were washed twice in ice-cold PBS. cDNA was prepared according to the manufacturer’s instructions (SMART-Seq^®^ v4 Ultra^®^ Low Input RNA Kit for sequencing). cDNA quality and quantity were verified using an Agilent Bioanalyzer (Santa Clara, CA, USA). For library preparation, 150 pg of cDNA was used per sample using the NexteraXT index kit. We performed quality control of our libraries using an Agilent Bioanalyzer and quantification with a qPCR kit (New England Biolabs). Libraries were then pooled to be subjected to next-generation sequencing in NextSeq550 for a paired-end 150 bp (2 × 75 bp) sequencing condition. Raw sequencing data can be found using the GEO accession numbers GSE300249 and GSE300250.

After performing quality control with FastQC v0.11.5, adapters were removed from the FASTQ files using Cutadapt v1.18 [22] with parameters -m 20 -O 6 -q 20. Reads were mapped to the reference genome mm10 (Mus Musculus GRCm38, Ensembl 102 Nov 2020) using STAR v2.7.0 [23], and BAM files were indexed using Samtools v1.14 [24]. Library complexity was estimated using Picard v2.26.7. Deeptools v3.5.1 [25] was used to transform BAM files to normalized BigWig files (with parameter--normalizeUsing CPM) to be visualized in the IGV [26] web app and to check for read enrichment in exons.

In R v4.2.0 and Bioconductor v3.15.2, the featureCounts function from Rsubread v2.10.5 [27] was used to count the number of reads in genes for each sample, using the Ensembl GTF annotation for the GRCm38 102 version and with the parameters GTF.featureType = “exon”, GTF.attrType = “gene_name”, useMetaFeatures = TRUE, isPairedEnd = TRUE, countReadPairs = TRUE, requireBothEndsMapped = TRUE, countMultiMappingReads = TRUE, and fraction = TRUE. The function filterByExpr from edgeR v3.38.1 [28] was used to keep only the genes with more than 5 counts in at least 4 samples and a minimum total count of 20. The count matrix was transformed and normalized using the voom function from limma v3.52.1 [29] and the quantile normalization method. Principal component analysis (PCA) was performed and plotted with FactoMineR v2.6 [30] after correcting the batch effect with the removeBatchEffect function from limma. Differential expression (DE) analysis between conditions was performed with limma including batch (and age in the case of HM cells) as covariates in the model and considering significance if the Benjamini–Hochberg adjusted *p*-value < 0.05. GSEA was performed on the t-statistic ordered DE gene list using clusterProfiler v4.4.1 [31], AnnotationDbi v1.58.0, and org.Mm.eg.db v3.15.0 with the gseGO function and parameters ont = “BP”, pvalueCutoff = 0.05, pAdjustMethod = “BH”, minGSSize = 10, maxGSSize = 500, seed = TRUE, and eps = 0, and the simplify function of clusterProfiler was used to reduce the redundancy of GO terms (where parameter cutoff = 0.7, by = “p.adjust”, select_fun = min). Significant GSEA results are represented in a bubble plot using Revigo [32], ggplot2 v3.3.6, and ggrepel v0.9.1. GSEA for several signatures was performed with the GSEA function of clusterProfiler and parameters minGSSize = 1, maxGSSize = Inf, pvalueCutoff = Inf, pAdjustMethod = “BH”, seed = TRUE, and eps = 0. ggplot2 was used to plot a lollipop chart of several significant HSPC signatures. The enrichment curves for the other signatures were plotted using the gseaplot2 function of enrichplot v1.16.1 and ggplot2. 

### 2.13. Statistical Analysis

Data analysis on the examined groups was performed using Graph Pad Prism 10.0. Data are reported as mean values ± SEM.

### 2.14. Ethical Compliance for Mouse Experiments

All mouse experiments at IDIBELL were performed in compliance with the ethical regulations according to the Spanish Law for Animal Protection and Welfare Code and were previously approved in the project CF19005/10805 (approval date: 11 March 2021) by IDIBELL’s Ethical Committee for Animal Experimentation (CEEA-IDIBELL) as well as by Generalitat of Catalunya. Mice breeding and maintenance at the British Columbia Cancer Research Centre (BCCRC), Terry Fox Laboratory, were carried out under the subject A24-0004 “Pre-clinical models of human blood malignancies” and approved by the Animal Care Committee of the University of British Columbia on 27 February 2026 (last renewal). The mice were housed according to the standards of the Canadian Council on Animal Care (CCAC).

### 2.15. Ethical Compliance for Patient-Derived Samples

NPM1c patient samples included in this study were collected in collaboration with the hematology unit of ICO, under the bioethical protocol PR211/21 approved by the Research Ethics Committee of the University Hospital of Bellvitge on 23 June 2021 from patients at first diagnosis (termed BCN). Samples were processed immediately and cryopreserved after the collection from the clinic. The storage and cryopreservation of human AML BM aspirates obtained at the Terry Fox Laboratory (BC Cancer Research Institute) by Dr. Kuchenbauers’s group (AML: H23-02637, initial approval on 22 September 2023) were performed by the hematology cell bank of British Columbia (BC). The experiments were approved by the UBC BC Cancer Research Ethics Board.

## 3. Results

### 3.1. Disruption of H3K9 Methylation in HSPCs Results in a Premature Aging Pre-Malignant Phenotype

**Figure 1:**
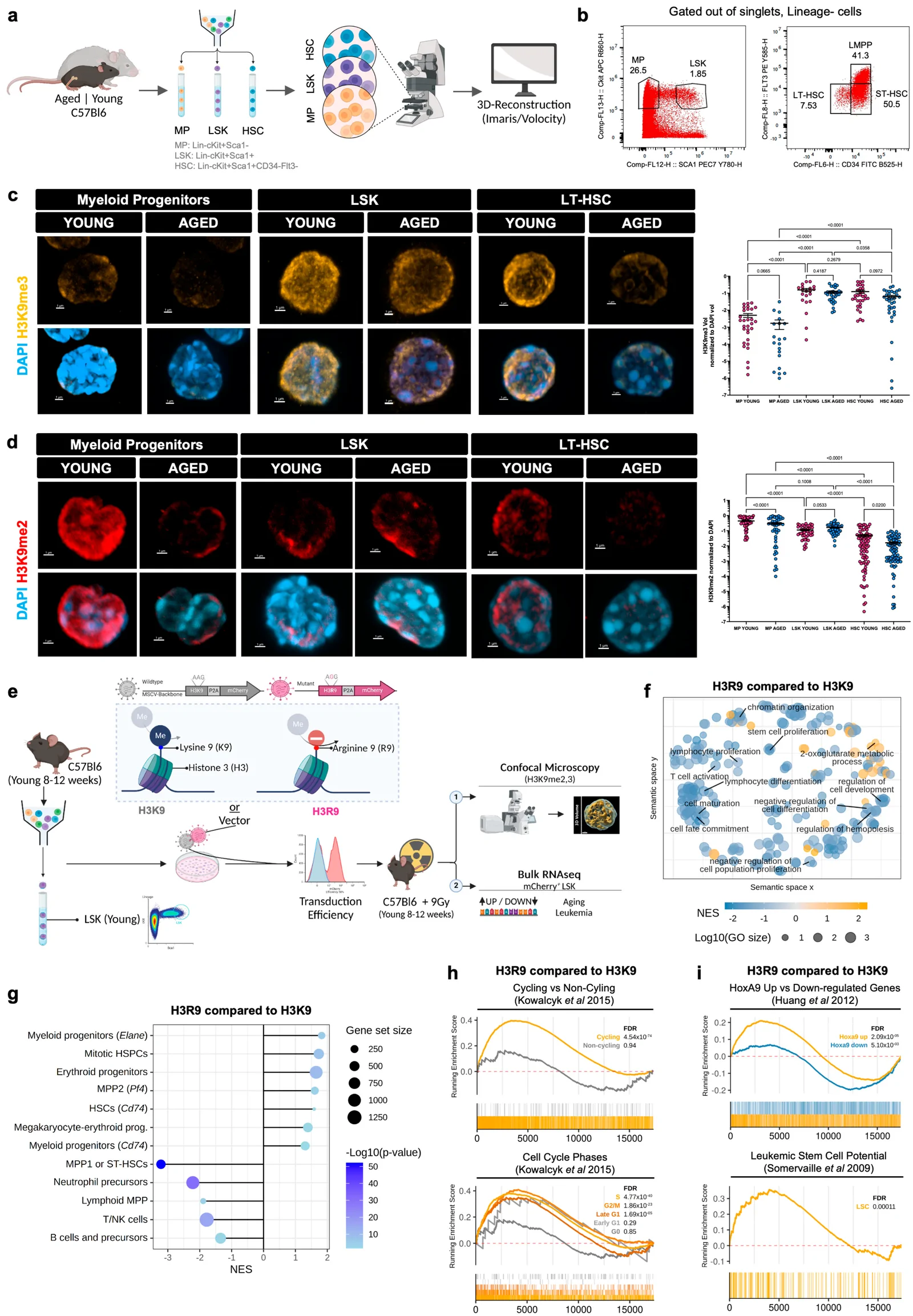
Disruption of H3K9 methylation in HSPCs results in a premature-aging pre-malignant phenotype. (**a**) Cartoon of the experimental set up for the 3D reconstruction of histone modifications in the sorted myeloid progenitor (MP: Lin-cKit+Sca1-), HSPCs (LSK: Lin-cKit+Sca1+), and HSC (Lin-cKit+Sca1+CD34-Flt3-) from the bone marrow of young (8–12 weeks) and aged (80–95 weeks) C57Bl/6 mice; (**b**) representative sorting gating strategy for MP, LSK, and HSCs in a young C57Bl/6 mouse; (**c**) representative 3D confocal microscopy images (extended focus plane) of young and aged MP, LSK, and HSCs stained with H3K9me3 (orange) and DAPI (blue). Scale bar: 1 µm. H3K9me3 volume was normalized to DAPI for quantification. n = 3 mice with 10–20 single cells per group. Data are plotted on a logarithmic scale and are presented as the mean ± SD, Kruskal–Wallis test; (**d**) representative 3D confocal microscopy images (extended focus plane) of young and aged MP, LSK, and HSCs stained with H3K9me2 (red) and DAPI (blue). Scale bar: 1 µm. H3K9me2 volume was normalized to DAPI for quantification. n = 3 mice with 15–30 single cells per group. Data are plotted on a logarithmic scale and are presented as the mean ± SD, Kruskal–Wallis test; (**e**) experimental layout. Young C57Bl/6 LSK samples were sorted and transduced with H3K9 (control, gray) or H3R9 (mutant, pink). Transduction efficiency tested by flow cytometry. Transduced cells (2 × 10^4^ cells/mouse) were transplanted into irradiated (9 Gy) young C57Bl/6 recipients. Confirmation of the reduction in H3K9 methylation by confocal microscopy (mCherry+ sorted BM) 12 weeks post-transplantation. Bulk RNA of transduced mCherry+ LSK was sequenced; (**f**) gene ontology (GO) terms obtained in the gene set enrichment analysis (GSEA) of H3R9 vs. H3K9 LSKs (Benjamini–Hochberg (BH) adjusted *p*-value < 0.05). NES: normalized enrichment score. GO size: size of the gene set. Selected GO terms are shown; (**g**) enrichment of significantly enriched HSPC gene signatures in H3R9 vs. H3K9–LSKs (BH adjusted *p*-value < 0.05) [34,35]; (**h**) Enrichment plot comparing H3R9 vs. H3K9 LSKs for cycling and non-cycling gene sets and for the cell cycle-phase gene sets [36]. Enriched in H3R9 (orange), decreased in H3R9 (blue), and not significant (gray); (**i**) enrichment plot comparing H3R9 vs. H3K9 LSKs for up- or downregulated HoxA9 targets [37] and LSC potential [38]. Colors as in (**h**). Figure created in BioRender.

H3K9 methylation is known to be involved in stem cell fate decision, and it is affected by aging and age-related malignancies [8,9,10,11]. First, to quantify H3K9 methylation in hematopoietic cells upon differentiation and aging, we sorted young (8–15-week-old C57BL/6 mice) and aged (85–95-week-old C57BL/6 mice) murine hematopoietic stem cells (gated as lineage-, c-Kit+, Sca-1+, CD34-, and Flt3-, and indicated as HSC), hematopoietic stem and progenitor cells (gated as lineage-, c-Kit+, and Sca-1+, and indicated as LSK), and myeloid progenitor cells (MP, gated as lineage-, c-Kit+, and Sca-1-), and analyzed H3K9me2 and H3K9me3 levels and localization in these cells by immunofluorescence followed by single-cell high-resolution 3D confocal microscopy analysis (Figure 1a,b). H3K9me3 marks constitutive heterochromatin and, as expected, is highly enriched at the nuclear periphery, next to the nuclear envelope, reflecting its heterochromatin localization [15,33] (Figure 1c). Three-dimensional reconstruction and quantification revealed higher H3K9me3 levels in LSK and HSCs compared to MPs (Figure 1c). It is important to note that H3K9me3 is slightly reduced in all aged samples compared to young samples, but the reduction is not significant, while H3K9me3 levels are significantly reduced in aged HSCs compared to aged LSK (Figure 1c). H3K9me2 is a more dynamically regulated histone modification that is enriched at facultative heterochromatin [15]. Interestingly, H3K9me2 levels were significantly elevated in MP cells compared to LSK and HSCs (Figure 1d). Our quantification also revealed clear age-related differences as aged MP and HSCs showed a decrease, relative to their young counterparts (Figure 1d). Overall, our data support an opposite change in H3K9 methylation pattern upon hematopoietic differentiation, with H3K9me3 levels decreasing and H3K9me2 levels increasing from HSCs down to myeloid commitment. Moreover, these results show a clear age-related disruption of H3K9me2, which decreases in aged HSCs and MP compared to young cells. 

Recently, by taking advantage of a retroviral H3K9 vector with an arginine substitution on position nine (H3K9→H3R9), which ablates any methylation on H3 at the K9 residue, we showed that reduced levels of H3K9me2 in HSCs functionally induce premature aging of HSCs and aging of the hematopoietic system [34]. To investigate further possible implications of reducing H3K9 methylation in HSPCs on gene expression, we retrovirally infected young LSK with the same H3R9 and control H3K9 vectors (tagged with mCherry) used previously [33] and transplanted the infected cells (Figure S1a) in lethally irradiated recipient mice. After 12 weeks from transplant, we performed confocal microscopy analysis and bulk RNA sequencing of mCherry+ LSKs sorted from H3K9 and H3R9 transplanted mice (Figure 1e). Of note, the arginine in position 9 prevents any methylation at this specific position leading to a global reduction in both me2 and me3 at H3K9 (Figure S1b). Principal component analysis (PCA) of RNA-seq data revealed that the H3K9 and H3R9 LSK cluster separated along the first dimension (Figure S1c). Despite H3K9 methylation being a repressive heterochromatin mark, its reduction resulted in a significant downregulation of multiple pathways, as revealed by Gene Set Enrichment Analysis (GSEA) of Gene Ontology (GO) biological processes (Figure 1f and Table S1). These pathways were predominantly associated with cell fate commitment, as well as lymphoid lineage proliferation and differentiation. Further analyses based on previously reported gene signatures showed myeloid skewing and a significant downregulation of lymphoid gene signatures in H3R9 LSK, supporting the premature aging phenotype (Figure 1g) [35,36]. In addition, H3R9 LSK shows significant enrichment for cell cycling gene signatures (Figure 1h) [37]. Given that myeloid expansion, impaired lymphoid differentiation, and enhanced proliferation and self-renewal are hallmark features of myeloid malignancies such as AML, we conducted GSEA to assess the enrichment of leukemic stem cell signatures and a myeloid disease signature comprising HoxA9 target genes, which are frequently dysregulated in AML [38,39]. We observed a significant positive enrichment of genes upregulated by HoxA9 and of leukemic stem cell signatures, indicating that the transcriptional changes induced by reduced H3K9 methylation resemble those associated with leukemogenic transformation (Figure 1i).

Together, these findings suggest that the aging-associated loss of H3K9 methylation contributes to a transcriptional program resembling leukemic transformation, characterized by myeloid bias, impaired lymphoid differentiation, and enhanced self-renewal, features shared by both aged HSCs and LSCs.

### 3.2. H3K9 Methylation Levels Are Altered in Leukemic Cells

**Figure 2:**
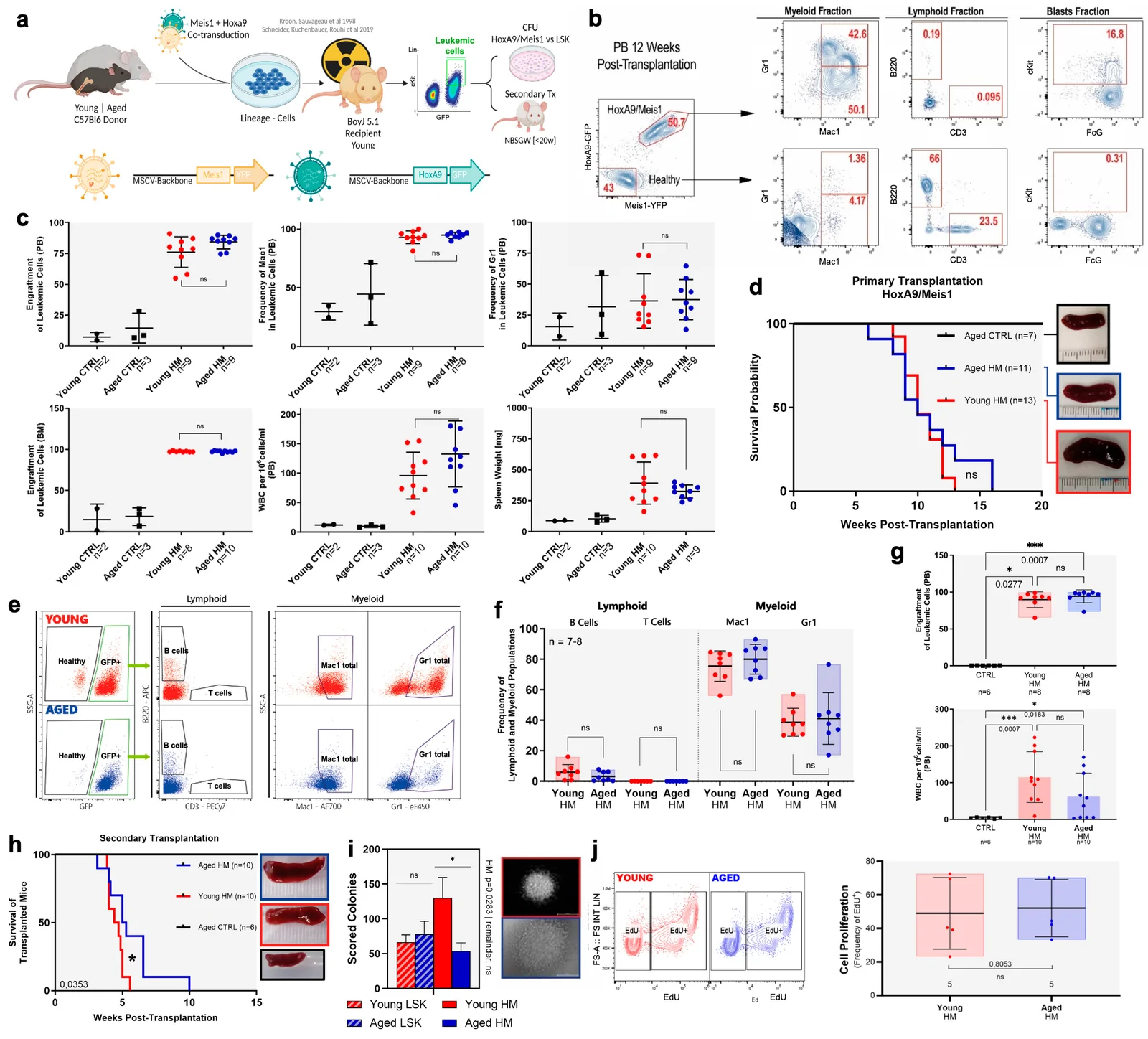
HoxA9/Meis1 overexpression in young and aged HSPCs induces AML in mice. (**a**) Cartoon scheme: lineage depleted cells were transduced with both HoxA9-GFP or Meis1-YFP (MSCV-background) and transplanted into irradiated (9 Gy) BoyJ Ly5.1 mice. BM cells were harvested and used for subsequent experiments (colony assay 1000 cells/condition, secondary transplantation 50 cells/mouse, confocal microscopy, transduction experiments) [19,41]; (**b**) flow cytometry analysis in the PB 12 weeks post-transplantation of HoxA9/Meis1 co-transduced cells; (**c**) cumulative data (9 weeks post-transplantation) of leukemic cell engraftments (in BM and PB; Lin-GFP+YFP+), Mac1+ and Gr1+ leukemic cells in PB, WBC in PB, and spleen weight. n = 8–10 (HM); 2–3 (Ctrl). Whiskers are mean ± SD. Mann–Whitney test (for contribution in PB), Welch’s *t*-test (for Mac1, Gr1, WBC, and spleen weight), and unpaired *t*-test (for engraftment in BM); (**d**) Kaplan–Meier survival curve of primary transplanted mice (black control vector, red young donor cells, blue aged donor cells) and respective exemplary pictures of spleen sizes, n = 7–13 mice/group. Log-rank (Mantel–Cox) test; (**e**) flow cytometric PB analysis 3–4 w post-secondary transplantation; (**f**) frequency of lymphoid and myeloid cells in PB 3–4 w post-secondary transplantation (when GFP > 50%). N = 7–8 mice/group. Whiskers are mean ± SD. Welch’s *t*-test (for B cells, T cells, and Gr1), unpaired *t*-test (for Mac1); (**g**) engraftment of leukemic cells and WBC counts in the PB 3–4 w post-secondary transplantation (when GFP > 50%); * *p* < 0.05, *** *p* < 0.001 (**h**) Kaplan–Meier survival curve of secondary transplanted NBSGW mice (control black, aged blue, young red). n = 6–10 mice/condition. Log-rank (Mantel–Cox) test; * *p* < 0.05 (**i**) colony assay of young LSK (red stripes, n = 2), aged LSK (blue stripes, n = 3), young HM (red, n = 11), and aged HM (blue, n = 9) cells. A total of 1000 cells were seeded in MethoCult medium and scored for total colony numbers after a range of 8–10 days. n = 4 independent repetitions (in duplicates) derived from different primary transplants. Whiskers are mean ± SEM. Kruskal–Wallis test with uncorrected Dunn’s test for multiple comparisons; * *p* < 0.05; (**j**) proliferation (EdU incorporation) assay of young (red) and aged (blue) leukemic cells (HMs). Representative cytometry scheme (left) and quantification (right). n = 5. Whiskers are mean ± SD. Welch’s *t*-test. Figure created in BioRender.

To investigate the function of H3K9 methylation in leukemogenesis and aging, we decided to establish young and aged HoxA9/Meis1 leukemic mouse models [40,41,42]. To this end, young and aged HSPCs (lineage-negative bone marrow cells) were co-transduced with *HoxA9* and *Meis1* overexpressing vectors and infected cells (HoxA9+/Meis1+, indicated from now on as HM cells) were transplanted into lethally irradiated young recipients (Figure 2a). At 12 weeks after transplantation, mice receiving HM cells showed an AML phenotype characterized by significant higher engraftment in BM and higher contribution to peripheral blood (PB), and compared to mice transplanted with young and aged control (empty vectors) cells, increased white blood count (WBC) and spleen weight, and myeloid skewing (Figure 2b,c). Both young and aged leukemic mice showed a similar phenotype and overall survival of a range of 3–4 months (Figure 2d). By a colony assay in vitro, we screened the expression of markers to enrich for leukemic initiating cells (Figure S2a). HM leukemic cells were lineage- (Cd11b-, Ter119-, Cd8-, Cd5-, B220-, Gr1-) and c-kit+, while all were Sca1- (Figure S2a). Furthermore, by flow cytometry we observed that all lineage-c-kit+ HM cells were negative for Il7Rα and CD150, and all were positive for FcγR and CD48 (Figure S2a). When comparing the colony-forming capacity of lineage-c-kit+, lineage-c-kit-, lineage+c-kit+, and lineage+c-kit- HM cells, lineage-c-kit+ HM cells grew clearly more colonies than all the other subsets in both young and aged samples (Figure S2b). To further dissect the leukemic potential of young and aged HM cells, we sorted the leukemic cells (lineage-c-kit+ HM) and performed secondary transplantation with 50 cells/mouse into non-irradiated NBSGW [43,44,45] (Figure 2a). These immunodeficient recipient mice are highly permissive for the engraftment of leukemic cells and do not require irradiation due to the hypomorphic c-kit mutation. The leukemic features of secondary transplanted recipients were comparable between mice receiving young or aged HM cells (Figure 2e–g). Notably, secondary recipients of young HM cells had a slightly but significantly shorter survival than their aged counterparts (average survival: 4.6 vs. 5.1 weeks; Figure 2h). In vitro, HM cells from young primary recipients demonstrated a greater proliferative capacity than those from aged mice, as assessed by colony-forming assays (Figure 2i), although EdU incorporation revealed no significant difference in proliferation rates between the two groups (Figure 2j).

**Figure 3:**
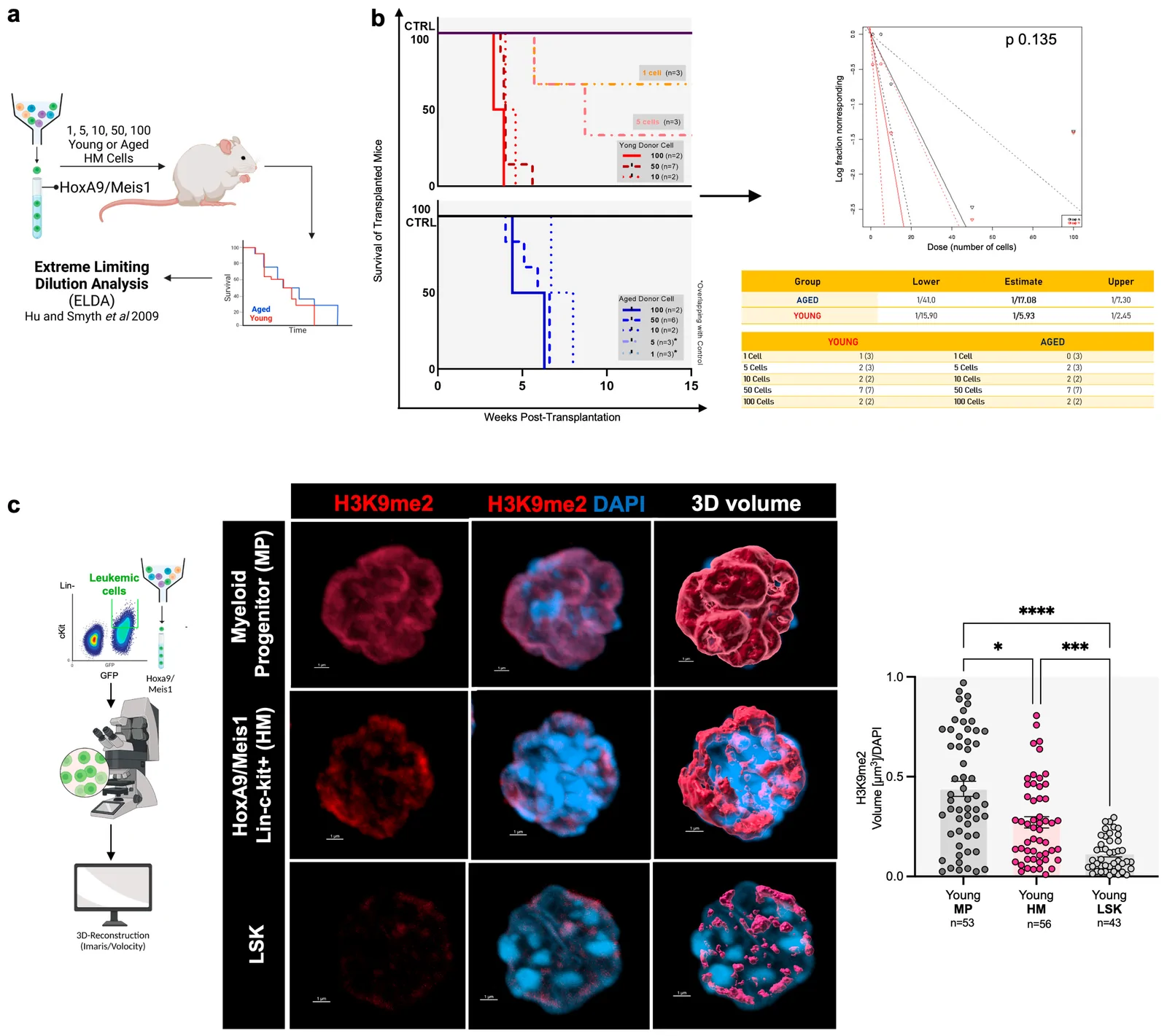
HoxA9/Meis1 AML development is not more aggressive for aged HM cells compared to young cells. (**a**) Cartoon scheme depicting the experimental set up [21]; (**b**) extreme limiting dilution assay (ELDA) of young (red) and aged (blue) donor cells. A total of 1 (n = 3), 5 (n = 3), 10 (n = 2), 50 (n = 6–7), and 100 cells (n = 2) were transplanted into NBSGW mice (8–12 w) and monitored for survival. Total numbers of mice per group are indicated as n; (**c**) cartoon scheme of the experimental set up. HoxA9/Meis1 cells obtained after primary transplantation were sorted and stained for confocal analysis. Representative images and 3D analysis of H3K9me2 in leukemic cells (HM, red), MP, and LSK (gray) normalized to DAPI volumes. Scale bar: 1 µm. n = 3 mice/experiment; 15–20 cells per experiment. Whiskers are mean ± SD. Kruskal–Wallis test with Dunn’s multiple comparisons test. * *p* < 0.05, *** *p* < 0.001, **** *p* < 0.0001. Figure created in BioRender.

To understand if the difference in leukemic stem cell (LSC) frequency between young and aged primary transplanted HM mice could account for the difference in survival and colony-forming capacity between samples, we performed a limiting dilution assay in vivo. We transplanted recipient mice with 1, 5, 10, 50, and 100 young and aged lineage-c-kit+ HM cells (Figure 3a). We estimated 1 LSC out of 17 lineage-c-kit+ HM cells in aged cells and 1 out of 6 in young cells, with no statistical differences between samples (Figure 3b).

To assess if H3K9 methylation levels are correlated to leukemogenesis, we sorted young and aged leukemic cells from primary transplanted mice and analyzed them by immunofluorescence followed by single-cell 3D quantification of H3K9me2. Both young and aged HM cells displayed similar levels of H3K9me2 (Figure S3a). Notably, H3K9me2 levels were significantly elevated in HM cells compared to healthy LSK cells but remained lower than those observed in MP cells (Figure 3c), suggesting that leukemic transformation is associated with an H3K9me2 level intermediate to that of the stem cells and the progenitors.

In conclusion, we successfully established young and aged *HoxA9/Meis1* overexpressing AML mouse models. Interestingly, leukemia development is not more severe or aggressive for aged HM cells compared to young cells. We even detected a significant increase in the colony-forming capacity of young leukemic cells compared to aged cells and a slight but significant increase in the mortality of secondary transplanted mice when receiving young HM cells compared to aged cells. It is important to note that while H3K9me2 is not different between young and aged leukemic cells, the levels of methylation in HM cells are significantly different from healthy HSPCs (higher in HM cells than in stem cells but lower in HM cells than in myeloid progenitor cells), supporting H3K9me2 as a possible target in AML independently from the chronological age of leukemic cells.

### 3.3. H3K9 Methylation in Leukemic Cells Is Linked to Their Proliferative Capacities

**Figure 4:**
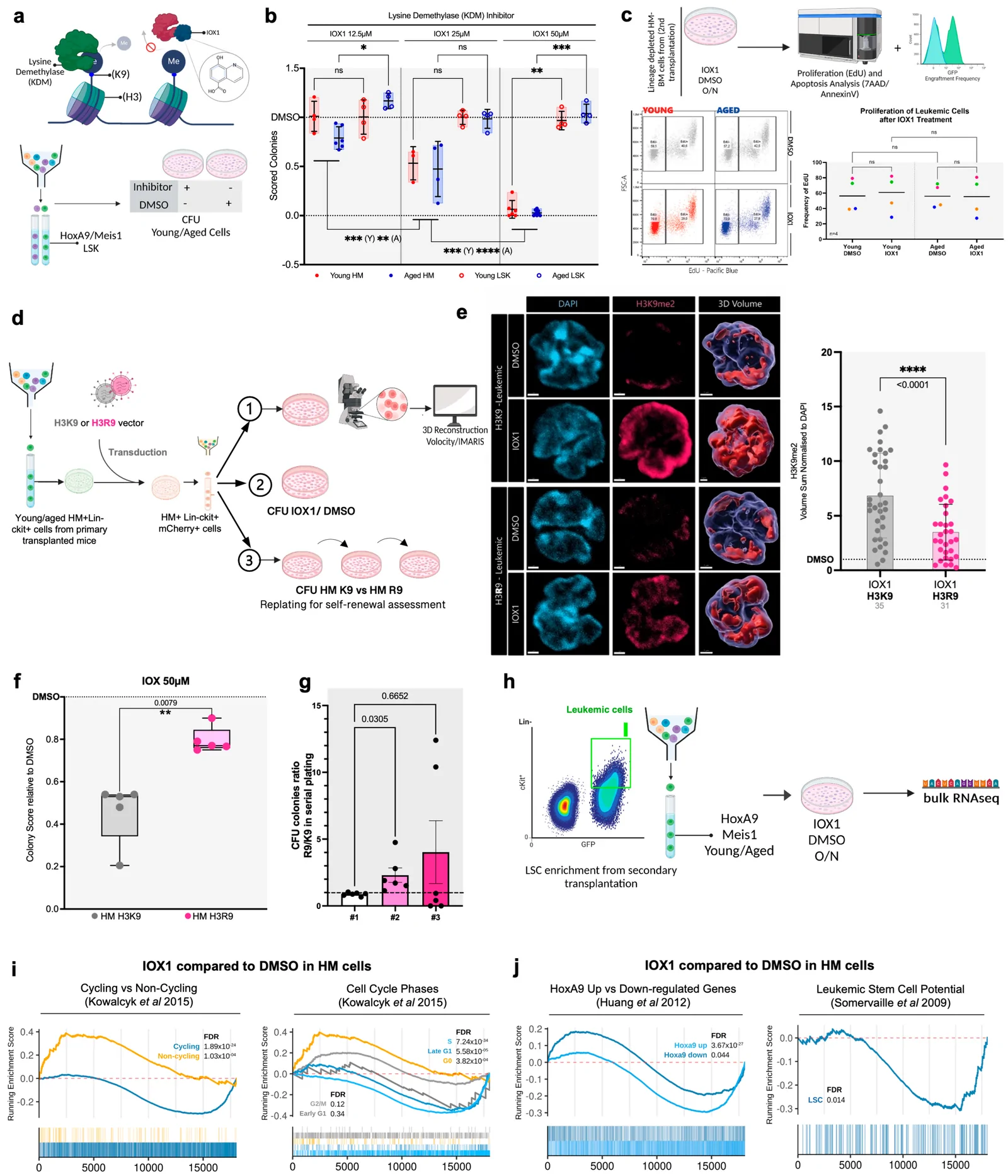
H3K9 methylation level in leukemic cells is linked to their proliferative capacities. (**a**) Mechanism of IOX1 inhibition and experimental design; (**b**) 1000 cells were seeded in MethoCult containing IOX1 (12.5 µM, 25 µM, and 50 µM) and total colonies were scored at a range of 7–10 d later. Young leukemic cells (HM cells; red), aged leukemic cells (HM cells; blue), young LSKs (red circle), and aged LSKs (blue circle). n = 3–9 repetitions. Whiskers are mean ± SD. Kruskal–Wallis test with uncorrected Dunn’s multiple comparisons test (for comparisons inside concentrations) and one-way ANOVA with Šídák’s multiple comparisons test (for comparisons across concentrations). All statistics between young and aged cells were ns; * *p* < 0.05, ** *p* < 0.01, *** *p* < 0.001, **** *p* < 0.0001. (**c**) proliferation assay of IOX1/DMSO (50 µM)-treated HM cells. Flow cytometric analysis of EdU incorporation after 20 h of treatment. n = 4 mice. Line is the median. Brown–Forsythe ANOVA test with Dunnett’s T3 multiple comparisons test; (**d**) experimental overview. Transduction of HM cells with H3R9 or H3K9 (both with mCherry reporter) and purified by sorting mCherry+ cells. Branch 1: cells were cultured with DMSO or IOX1 50 µM (up to 16 h), stained for H3K9me2, and 3D reconstructed. Branch 2: cultured in a colony assay with DMSO or IOX1 50 µM. Branch 3: cultured for serial CFU plating for self-renewal assessment in the absence of IOX1 (Anova, Kruskal–Wallis); (**e**) representative images of transduced murine HM cells (H3K9 wild type; H3R9 mutant) stained for H3K9me2 (red) and DAPI (blue). Quantification of H3K9me2 3D volumes normalized to DAPI volumes. To plot the data, single IOX1 points were normalized to the mean DMSO value. n = 2 mice/group. Whiskers are mean ± SD. Welch’s *t*-test; **** *p* < 0.0001; (**f**) colony assay of transduced cells scored after a range of 10–12 days. HM leukemic (HM, gray), transduced control (H3K9, gray), and mutant (H3R9, pink) cells. n = 3–6. Whiskers are mean ± SD. Mann–Whitney test; ** *p* < 0.01. (**g**) CFU assay in serial re-plating for self-renewal assessment of HM leukemic cells transduced with H3K9 or HR9 retroviral vectors. Number of colonies from H3R9 transduced cells is normalized over the number of colonies from H3K9 transduced cells obtained for the same HM leukemic mouse sample. (**h**) HM cells derived from primary transplantations were enriched (Lin-cKit+GFP+) and cultured for 16 h with 50 µM IOX1/DMSO and processed for bulk RNA-seq; (**i**) enrichment plot comparing IOX1 vs. DMSO treatment in HM cells for cycling and non-cycling gene sets (**left**) and for the cell cycle-phase gene sets (**right**). Colors as in 1 h [37]; (**j**) enrichment plot comparing IOX1 vs. DMSO treatment in HM cells for up- or downregulated HoxA9 targets [38] (**left**) and LSC-potential signature (**right**) [39]. Figure created in BioRender.

To further explore if H3K9 methylation could be a potential target for decreasing leukemic cell proliferation, we performed colony assays with HM cells treated with IOX1 (8-hydroxyquinoline-5-carboxylic acid), which mimics 2-OG (α-KG) and blocks the catalytic activity of lysine demethylases [46,47] (resulting in H3K9 methylation increase). In parallel, we assessed the colony-forming capacity of treated young and aged healthy LSK cells as a control (Figure 4a). IOX1 selectively and dose-dependently suppressed the colony-forming ability of both young and aged HM cells, with no detectable effect on healthy LSKs (Figure 4b). Notably, treatment with 50 μM IOX1 nearly abolished colony formation in HM cells. This effect is consistent with previous findings linking lysine demethylase activity to key cellular processes such as proliferation and apoptosis [12,13,14]. However, short-term treatment with IOX1 (24 h) did not significantly impact proliferation or apoptosis in leukemic HM cells as well as in healthy non-leukemic HSPCs (Figure 4c and Figure S3b,c), suggesting that its inhibitory effect on colony formation may reflect impaired proliferative capacity rather than acute cytotoxicity. 

To directly assess the impact of H3K9 methylation on HM cell proliferative capacity, we infected HM cells with the H3R9 mutant construct, alongside the H3K9 wild-type control vector. Next, we cultured H3R9/H3K9 HM cells with IOX1 and analyzed them by confocal microscopy and colony-forming assays (Figure 4d). Quantification using single-cell 3D confocal analysis revealed a significant increase in H3K9me2 levels upon IOX1 treatment (consistent with its reported lysine demethylase inhibitor activity) in H3K9 HM cells, while levels of H3K9me2 remained significantly lower in H3R9 HM cells (Figure 4e). IOX1 might also affect methylation at other lysines on histone 3, and by the quantification of 3D IF confocal images we detected an increase in H3K4me3, a decrease in H3K27me3, and no changes in H3K36me3 after IOX1 treatment (Figure S3d). Notably, reduced H3K9me2 correlated with the significantly diminished ability of IOX1 to inhibit colony formation in H3R9 cells, and colony assays confirmed the rescue of clonogenicity in IOX1-treated H3R9 HM cells relative to treated H3K9 HM cells (Figure 4f), supporting the conclusion that IOX1’s inhibitory effect on HM cells is at least partly dependent on increased H3K9 methylation. 

Next, to test whether low levels of H3K9 methylation in leukemic cells are required to maintain self-renewal, we performed serial CFU re-plating of HM cells transduced with H3K9 or H3R9 retroviral vectors (Figure 4d, branch 3). We analyzed the number of colonies in H3R9 in H3K9 HM samples (Figure 4g). Data show that H3R9 overexpressing leukemic cells form significantly more colonies at second re-plating and show a non-significant increase in self-renewal capacity upon third re-plating (Figure 4g). Overall, we cannot conclude that low levels of H3K9 methylation increase the self-renewal capacity of HM leukemic cells. Technical factors might play a role, like sporadic silencing of the transgene in long-term culturing experiments, which has been reported by other investigators in the past [48,49]. Moreover, we cannot exclude the differences in the long-term clonal capacity between different donors, as has already been shown for retroviral-induced HM leukemia [50]. 

Furthermore, to gain insight into the molecular changes induced by IOX1 treatment in HM cells, we performed bulk RNA sequencing on HM cells treated with IOX1 or DMSO as a control (Figure 4h and Table S2). IOX1 treatment resulted in a marked downregulation of cell cycle-related gene signatures, alongside a reduced expression of genes typically upregulated by HoxA9 and those associated with high leukemic stem cell potential (Figure 4i,j) [37,38,39]. 

Together, these findings establish a functional link between H3K9 methylation levels and leukemic proliferative capacity and identify H3K9 methylation regulation as a potential epigenetic vulnerability in LSCs.

### 3.4. IOX1 Treatment Targets LSCs in NPM1mut AML Patients

**Figure 5:**
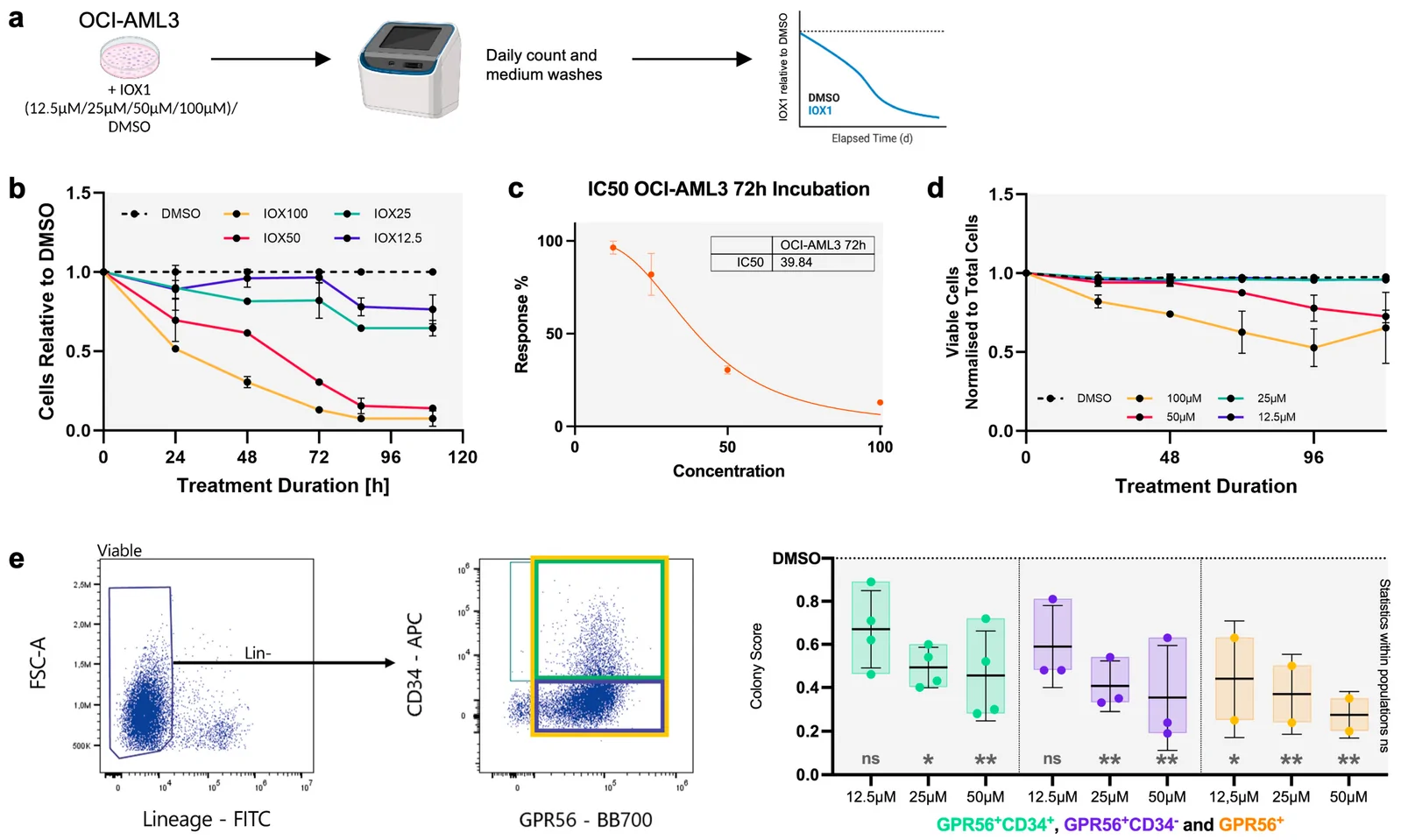
Targeting H3K9 methylation decreases the proliferation of human leukemic cells. (**a**) Cartoon scheme: OCI-AML3 and other human cell lines were cultured in DMSO or IOX1 (12.5 µM, 25 µM, 50 µM, or 100 µM) over 6 consecutive days. Cells were counted every day accompanied by daily medium changes; (**b**) growth curves of SKM-1, UCSD-AML1, and SKNO-1 treated with IOX1 and shown normalized to DMSO; (**c**) IC50 of all tested cell lines at 72 h of treatment. Concentration is indicated in a table; (**d**) growth curves of OCI-AML3 under different IOX1 concentrations and normalized to DMSO (left) and cell viability over time of OCI-AML3 under different concentrations of IOX1 or DMSO (right); (**e**) LSCs from human primary BM NPM1c-AML samples were enriched using CD34-/CD34+GPR56+ (blue and green frames) or GPR56+ (yellow frame) populations. A total of 1000 cells were assessed in a colony assay for 14 days with IOX1 or DMSO. The summarized data are shown normalized to DMSO, statistics comparing to DMSO are depicted below the columns, and statistics within groups are all ns. n = 2–4 individual BM donors; each experiment was performed in duplicates. Whiskers are means ± SD. Kruskal–Wallis with uncorrected Dunn’s test (for comparisons to DMSO) and with Dunn’s multiple comparisons test (for comparisons within groups). * *p* < 0.05, ** *p* < 0.01, ns, not significant. Figure created in BioRender.

To evaluate the therapeutic potential of targeting H3K9 methylation in human AML, we first assessed the effect of IOX1 across a panel of AML cell lines with diverse genetic backgrounds (Figure 5a). All tested lines exhibited a dose-dependent reduction in proliferation following IOX1 treatment (Figure 5b), with most showing 50% growth inhibition at concentrations of 50 μM or lower after 72 h (Figure 5c). SKM-1 was the only line requiring higher doses (Figure 5c).

Among these, OCI-AML3, a cell line harboring the *NPM1c* mutation and overexpressing *HOXA9/MEIS1*, showed marked sensitivity to IOX1 (Figure 5c). The treatment significantly impaired proliferation and dose-dependently increased H3K9me2,3 levels, consistent with the inhibition of lysine demethylase activity through IOX1 (Figure 5d and Figure S4a,b). Of note, cell viability remained largely unaffected, even at higher IOX1 concentrations (50–100 μM; Figure 5d).

To extend these findings to a clinically relevant setting, we analyzed primary bone marrow samples from *NPM1*mut AML patients. Aspirates were obtained at diagnosis from patients aged 64–80 years (with equal male and female representation), and their clinical data are summarized in Table S3. As *NPM1*mut AML cells often lack CD34 expression [51], we used GPR56 as an alternative marker to enrich for LSC (Figure S4b) [52,53]. Due to the rapid in vivo metabolism of IOX1 [46], ex vivo colony-forming assays were used to assess proliferation capacity. IOX1 treatment led to a robust, dose-dependent reduction in colony formation across all *NPM1*mut samples, independent of CD34 status (Figure 5e).

In summary, IOX1 effectively impairs the proliferative capacity of LSC in *NPM1*mut AML patient samples. These results highlight H3K9 methylation regulation as a potential therapeutic vulnerability in this highly prevalent AML subtype.

## 4. Discussion

Recently, we have shown that decreased levels of H3K9 methylation in HSCs induce a premature aging-like phenotype [34]. In the current study, we further demonstrate that alterations in H3K9 methylation in murine HSPCs contribute to a pre-leukemic phenotype, independently of chronological age. Previous studies have reported age-related changes in H3K9 methylation-modifying enzymes [10,33,54], and the deregulation of H3K9 methylation has been linked to impaired chromosomal stability and an elevated risk of leukemogenesis [55]. More recently, it has also been shown that H3K9 methylation represents a target to eliminate chronic myelomonocytic leukemia stem cells [33]. 

Here, we observe a decrease in H3K9me2 in healthy murine, aged MP and HSCs compared to young samples (Figure 1d). Transcriptomic analysis of LSK cells lacking H3K9 methylation (H3R9 LSK) identified significant alterations in HoxA9 target genes, indicating heightened HoxA9 activity in the absence of H3K9 methylation. This deregulation of HoxA9, which frequently occurs in leukemic cells [56,57], was accompanied by an enrichment of cycling gene signatures and gene signatures associated with leukemic potential, further supporting the role of H3K9 methylation as a regulator of genomic stability [58]. Despite these findings, key questions remain, such as whether low H3K9 methylation levels act as a driver in leukemogenesis and whether they are essential for the maintenance of leukemic cells. Moreover, it would be of interest to identify the exact chromatin and transcriptional targets of H3K9 methylation inhibiting leukemic transformation in HSPCs. However, H3K9 methylation has been previously reported to bind mainly at heterochromatin and at repetitive regions of the genome, and its removal does not induce directly gene transcription, which requires other chromatin modifications and transcription factors [15,16,34]. Therefore, it is not possible in the current study to establish a more direct link between the changes in H3K9 methylation and the transcriptome in HSPCs.

To investigate the role of H3K9 methylation in murine AML, we induced leukemia using *HoxA9/Meis1* (HM) overexpression, which on the one hand reflects clinically relevant alterations identified in both young and elderly AML patients, and, on the other hand, was found to be significantly affected in our H3K9→R9 system (HoxA9 target gene upregulation). The results show that young HM cells did not exhibit faster disease progression, and our data do not support more aggressive leukemic progression in chronologically aged cells compared to young. Interestingly, both young and aged HM cells showed lower levels of H3K9me2 compared to fully differentiated MPs and higher H3K9me2 levels relative to LSK cells, suggesting that increased H3K9me2 does not linearly correspond to a global increase in transcriptional repression. Supporting this notion, prior studies have demonstrated that while H3K9me2 levels are elevated in AML, chromatin remains uncondensed. Instead, H3K9me2 accumulates at specific loci, including genes downregulated in AML and at sites of AML-related chromosomal translocations [59].

It is noteworthy that our data demonstrate that increasing H3K9 methylation by IOX1 treatment, which inhibits lysine demethylases, selectively reduces the proliferative potential of leukemic cells, including murine and patient-derived AML samples as well as AML cell lines. Studies have shown that lysine demethylases, such as KDM4A, are frequently altered in cancer, providing a plausible explanation for the elevated H3K9 methylation levels observed and the efficacy of IOX1 in targeting leukemic cells [60]. Moreover, genetic manipulation of H3K9 methylation levels revealed that maintaining low levels of H3K9 methylation is critical for leukemic cell proliferation in CFU assays (Figure 4g). Considering that IOX1 treatment on HM leukemic cells has no effect on cell proliferation and apoptosis (Figure 4c and Figure S3), and that treatment with IOX1 of HM leukemic cells overexpressing the H3R9 variant rescues their clonogenic capacity in the CFU assay (Figure 4f), we conclude that IOX1 leads to a decreased proliferative capacity of HM leukemic cells, at least in part by increasing H3K9 methylation. Consistently, the data show that IOX1 treatment on leukemic cells leads to increased H3K9 methylation levels (Figure 4e), a decrease in HoxA9 and cycling gene signatures, and downregulation of genes associated with leukemic potential (Figure 4i,j).

## 5. Conclusions

Treating HoxA9-Meis1 leukemic cells with IOX1 to increase H3K9 methylation levels induces a decrease in HoxA9 and cycling gene signatures, and a downregulation of genes associated with leukemic potential. Moreover, IOX1 treatment selectively reduces the proliferative potential of leukemic cells, both from murine and patient-derived AML samples, as well as of AML cell lines. Collectively, our findings suggest that targeting H3K9 methylation may offer a therapeutic strategy for AML, which is independent of the patient’s genetic background, the proliferative state of leukemic stem cells, and age.

## Data Availability

The source data for Figure 1, Figure 2, Figure 3, Figure 4 and Figure 5 and Supplementary Figures S1–S4 are provided as Supplementary Materials. RNA-seq data are deposited at GEO (accession number GSE344936) and the code used to analyze the data can be directed to the corresponding author. Dilutions and catalogue numbers of all commercial antibodies are provided in the Supplementary Materials.

## Supplementary Materials

The following supporting information can be downloaded at: https://www.mdpi.com/article/10.3390/cancers18182969/s1, **Figure S1**. H3K9>R9 infected cells show reduced levels of H3K9 methylation. (**a**) Representative flow cytometry analysis of mCherry+ BM cells infected with H3K9 and H3R9 vectors; (**b**) Representative 3D confocal images of young MPs and young LSKs infected with H3K9 and H3R9 vectors, sorted and stained for H3K9me2 and H3K9me3 quantification. Whiskers show mean ± SD. Mann-Whitney test; (**c**) PCA of H3R9 (pink) and H3K9 (grey) LSK’s RNA-seq data. Normalized read counts in genes per sample; **Figure S2**. Characterization of HoxA9/Mesi1 leukemic cells. (**a**) Flow cytometry analysis of HoxA9/Meis1+ leukemic bone marrow cells shows that all cells are Sca1-, while Sca1 is expressed by HoxA9/Meis1- bone marrow cells from the same mouse. All HoxA9/Meis1+ leukemic bone marrow cells are Fcγ+, CD48+, CD150- and IL7Rα-; (**b**) Cartoon of the gating scheme and of the serial plating experiment performed in vitro to identify the cell population enriched for LIC. The bar graph shows the number of colonies obtained by each subpopulation of young and aged leukemic cells. Representative images of the colonies are shown on the right side. HoxA9/Meis1+ Lin-c-kit+ cells are enriched for LIC; **Figure S3**. IOX1 treatment does not increase apoptosis in young and aged HM cells. (**a**) 3D-analysis of H3K9me2 in leukemic cells (young HM, red and aged HM, blue) normalised to DAPI-volume. n = 3 mice/experiment, 15–20 cells per experiment. Whiskers are mean ± SD. Non parametric *t*-test. (**b**) Representative flow cytometry analysis and quantification of HoxA9/Meis1+ AnnexinV+ leukemic bone marrow cells after 20 h of IOX1 treatment. Controls were treated with the solvent (DMSO). Whiskers are mean ± SD. One-way ANOVA with Sídák’s multiple comparisons test. (**c**) Representative flow cytometry analysis and quantification of HSPCs (Lineage- bone marrow cells) AnnexinV+ after 20 h of IOX1 treatment (50 µM and 200 µM). Controls were treated with the solvent (DMSO). Whiskers are mean ± SD. One-way ANOVA with Sídák’s multiple comparisons test. (**d**) Representative 3D confocal images of young HSCs treated for 14–16 h with +/-IOX1 50 µM and stained for H3K4me3, H3K27me3 and H3K36me3 quantification. Whiskers show mean ± SD. Mann-Whitney test. ** *p* < 0.01, *** *p* < 0.001; **Figure S4**. (**a**) Representative western blot analysis of H3K9me2 and H3K9me3 in OCI-AML3+/- 50 µM IOX1 treatment at different time points. (**b**) Quantification of the western blot analysis of H3K9me2 and H3K9me3 in OCI-AML3+/- 50 µM and 200 µM IOX1 treatment at 20 h. (**c**) Representative sorting strategy of human NPM1mut AML cells stained with lineage markers, CD34 and GPR56. The graphs show the engraftment of human donor-derived CD45+ cells and the frequency of human GPR56+ and CD33+ cells among human donor-derived CD45+ cells in xenotransplanted mice 8 weeks after transplant. Whiskers show mean ± SD. Survival of xenotransplanted mice is also shown. Kruskal-Wallis test with uncorrected Dunn’s test for multiple comparisons. Log-rank (Mantel-Cox) test for survival analysis. * *p* < 0.05. Figure created in BioRender; **Table S1**. Data for the RNA-seq experiment on H3K9 and H3R9 young LSKs. Information on the samples, the differentially expressed genes, and the GSEA; **Table S2**. Data for the RNA-seq experiment on IOX1-treated and DMSO-treated HM cells. Information on the samples and the differentially expressed genes; **Table S3**. NMP1 AML patient cohort data.

## Abbreviations

The following abbreviations are used in this manuscript:

HSPCs	Hematopoietic stem and progenitor cells
AML	Acute myeloid leukemia
H3K9 methylation	Histone 3 lysine 9 methylation
PTMs	Post-translational modifications
HSCs	Hematopoietic stem cells
BM	Bone marrow
me2	Dimethylated
me3	Trimethylated
LSCs	Leukemic stem cells
LSKs	Lin- cKit+ Sca1+ bone marrow cells
MPs	Myeloid progenitors
H3K9	Histone 3 lysine 9
H3R9	Histone 3 arginine 9
DAPI	4′,6-diamidino-2-phenylindole
SEM	Standard error of mean
RNA	Ribonucleic acid
PCA	Principal component analysis
GSEA	Gene set enrichment analysis
GO	Gene ontology
NES	Normalized enrichment score
BH	Benjamini–Hochberg
HMs	HoxA9+/Meis1+ cells
PB	Peripheral blood
WBCs	White blood cells
SD	Standard deviation
GFP	Green fluorescent protein
IOX1	8-hydroxyquinoline-5-carboxylic acid
DMSO	Dimethyl sulfoxide

## References

[1] Rodriguez-Fraticelli A.E., Wolock S.L., Weinreb C.S., Panero R., Patel S.H., Jankovic M., Sun J., Calogero R.A., Klein A.M., Camargo F.D. (2018). Clonal analysis of lineage fate in native haematopoiesis. Nature.

[2] Velten L., Haas S.F., Raffel S., Blaszkiewicz S., Islam S., Hennig B.P., Hirche C., Lutz C., Buss E.C., Nowak D. (2017). Human haematopoietic stem cell lineage commitment is a continuous process. Nat. Cell Biol..

[3] Beerman I., Bhattacharya D., Zandi S., Sigvardsson M., Weissman I.L., Bryder D., Rossi D.J. (2010). Functionally distinct hematopoietic stem cells modulate hematopoietic lineage potential during aging by a mechanism of clonal expansion. Proc. Natl. Acad. Sci. USA.

[4] Dykstra B., Olthof S., Schreuder J., Ritsema M., de Haan G. (2011). Clonal analysis reveals multiple functional defects of aged murine hematopoietic stem cells. J. Exp. Med..

[5] Dong Y., Shi O., Zeng Q., Lu X., Wang W., Li Y., Wang Q. (2020). Leukemia incidence trends at the global, regional, and national level between 1990 and 2017. Exp. Hematol. Oncol..

[6] Bonnet D., Dick J.E. (1997). Human acute myeloid leukemia is organized as a hierarchy that originates from a primitive hematopoietic cell. Nat. Med..

[7] Chung Y.R., Schatoff E., Abdel-Wahab O. (2012). Epigenetic alterations in hematopoietic malignancies. Int. J. Hematol..

[8] Grigoryan A., Guidi N., Senger K., Liehr T., Soller K., Marka G., Vollmer A., Markaki Y., Leonhardt H., Buske C. (2018). LaminA/C regulates epigenetic and chromatin architecture changes upon aging of hematopoietic stem cells. Genome Biol..

[9] Montavon T., Shukeir N., Erikson G., Engist B., Onishi-Seebacher M., Ryan D., Musa Y., Mittler G., Meyer A.G., Genoud C. (2021). Complete loss of H3K9 methylation dissolves mouse heterochromatin organization. Nat. Commun..

[10] Djeghloul D., Kuranda K., Kuzniak I., Barbieri D., Naguibneva I., Choisy C., Bories J.-C., Dosquet C., Pla M., Vanneaux V. (2016). Age-Associated Decrease of the Histone Methyltransferase SUV39H1 in HSC Perturbs Heterochromatin and B Lymphoid Differentiation. Stem Cell Rep..

[11] Arifuzzaman S., Khatun M.R., Khatun R. (2020). Emerging of lysine demethylases (KDMs): From pathophysiological insights to novel therapeutic opportunities. Biomed. Pharmacother..

[12] Nicetto D., Zaret K.S. (2019). Role of H3K9me3 heterochromatin in cell identity establishment and maintenance. Curr. Opin. Genet. Dev..

[13] Olcina M.M., Leszczynska K.B., Senra J.M., Isa N.F., Harada H., Hammond E.M. (2016). H3K9me3 facilitates hypoxia-induced p53-dependent apoptosis through repression of APAK. Oncogene.

[14] Nicetto D., Donahue G., Jain T., Peng T., Sidoli S., Sheng L., Montavon T., Becker J.S., Grindheim J.M., Blahnik K. (2019). H3K9me3-heterochromatin loss at protein-coding genes enables developmental lineage specification. Science.

[15] Padeken J., Methot S.P., Gasser S.M. (2022). Establishment of H3K9-methylated heterochromatin and its functions in tissue differentiation and maintenance. Nat. Rev. Mol. Cell Biol..

[16] Methot S.P., Padeken J., Brancati G., Zeller P., Delaney C.E., Gaidatzis D., Kohler H., van Oudenaarden A., Großhans H., Gasser S.M. (2021). H3K9me selectively blocks transcription factor activity and ensures differentiated tissue integrity. Nat. Cell Biol..

[17] Kühn M.W.M., Song E., Feng Z., Sinha A., Chen C.-W., Deshpande A.J., Cusan M., Farnoud N., Mupo A., Grove C. (2016). Targeting Chromatin Regulators Inhibits Leukemogenic Gene Expression in NPM1 Mutant Leukemia. Cancer Discov..

[18] Wang F., Travins J., DeLaBarre B., Penard-Lacronique V., Schalm S., Hansen E., Straley K., Kernytsky A., Liu W., Gliser C. (2013). Targeted Inhibition of Mutant IDH2 in Leukemia Cells Induces Cellular Differentiation. Science.

[19] Schneider E., Staffas A., Röhner L., Malmberg E.D., Ashouri A., Krowiorz K., Pochert N., Miller C., Wei S.Y., Arabanian L. (2018). Micro-ribonucleic acid-155 is a direct target of Meis1, but not a driver in acute myeloid leukemia. Haematologica.

[20] Yu Y., Schleich K., Yue B., Ji S., Lohneis P., Kemper K., Silvis M.R., Qutob N., van Rooijen E., Werner-Klein M. (2018). Targeting the Senescence-Overriding Cooperative Activity of Structurally Unrelated H3K9 Demethylases in Melanoma. Cancer Cell.

[21] Hu Y., Smyth G.K. (2009). ELDA: Extreme limiting dilution analysis for comparing depleted and enriched populations in stem cell and other assays. J. Immunol. Methods.

[22] Martin M. (2011). Cutadapt removes adapter sequences from high-throughput sequencing reads. EMBnet. J..

[23] Dobin A., Davis C.A., Schlesinger F., Drenkow J., Zaleski C., Jha S., Batut P., Chaisson M., Gingeras T.R. (2013). STAR: Ultrafast universal RNA-seq aligner. Bioinformatics.

[24] Danecek P., Bonfield J.K., Liddle J., Marshall J., Ohan V., Pollard M.O., Whitwham A., Keane T., McCarthy S.A., Davies R.M. (2021). Twelve years of SAMtools and BCFtools. GigaScience.

[25] Ramírez F., Ryan D.P., Grüning B., Bhardwaj V., Kilpert F., Richter A.S., Heyne S., Dündar F., Manke T. (2016). deepTools2: A next generation web server for deep-sequencing data analysis. Nucleic Acids Res..

[26] Thorvaldsdóttir H., Robinson J.T., Mesirov J.P. (2013). Integrative Genomics Viewer (IGV): High-performance genomics data visualization and exploration. Brief. Bioinform..

[27] Liao Y., Smyth G.K., Shi W. (2019). The R package Rsubread is easier, faster, cheaper and better for alignment and quantification of RNA sequencing reads. Nucleic Acids Res..

[28] Robinson M.D., McCarthy D.J., Smyth G.K. (2010). edgeR: A Bioconductor package for differential expression analysis of digital gene expression data. Bioinformatics.

[29] Ritchie M.E., Phipson B., Wu D., Hu Y., Law C.W., Shi W., Smyth G.K. (2015). limma powers differential expression analyses for RNA-sequencing and microarray studies. Nucleic Acids Res..

[30] Le S., Josse J., Husson F. (2008). FactoMineR: An R Package for Multivariate Analysis. J. Stat. Softw..

[31] Wu T., Hu E., Xu S., Chen M., Guo P., Dai Z., Feng T., Zhou L., Tang W., Zhan L. (2021). clusterProfiler 4.0: A universal enrichment tool for interpreting omics data. Innovation.

[32] Supek F., Bošnjak M., Škunca N., Šmuc T. (2011). REVIGO Summarizes and Visualizes Long Lists of Gene Ontology Terms. PLoS ONE.

[33] Hidaoui D., Porquet A., Chelbi R., Bohm M., Polyzou A., Alcazer V., Depil S., Imanci A., Morabito M., Renneville A. (2024). Targeting heterochromatin eliminates chronic myelomonocytic leukemia malignant stem cells through reactivation of retroelements and immune pathways. Commun. Biol..

[34] Mejía-Ramírez E., Picazo P.I., Walter B., Montserrat-Vazquez S., Affuso F., Wieser S., Pezzano F., Reymond L., Castillo-Robles J., Matteini F. (2026). Targeting RhoA nuclear mechanoactivity rejuvenates aged hematopoietic stem cells. Nat. Aging.

[35] Montserrat-Vazquez S., Ali N.J., Matteini F., Lozano J., Zhaowei T., Mejia-Ramirez E., Marka G., Vollmer A., Soller K., Sacma M. (2022). Transplanting rejuvenated blood stem cells extends lifespan of aged immunocompromised mice. npj Regen. Med..

[36] Velten L., Story B.A., Hernández-Malmierca P., Raffel S., Leonce D.R., Milbank J., Paulsen M., Demir A., Szu-Tu C., Frömel R. (2021). Identification of leukemic and pre-leukemic stem cells by clonal tracking from single-cell transcriptomics. Nat. Commun..

[37] Kowalczyk M.S., Tirosh I., Heckl D., Rao T.N., Dixit A., Haas B.J., Schneider R.K., Wagers A.J., Ebert B.L., Regev A. (2015). Single-cell RNA-seq reveals changes in cell cycle and differentiation programs upon aging of hematopoietic stem cells. Genome Res..

[38] Huang Y., Sitwala K., Bronstein J., Sanders D., Dandekar M., Collins C., Robertson G., MacDonald J., Cezard T., Bilenky M. (2012). Identification and characterization of Hoxa9 binding sites in hematopoietic cells. Blood.

[39] Somervaille T.C.P., Matheny C.J., Spencer G.J., Iwasaki M., Rinn J.L., Witten D.M., Chang H.Y., Shurtleff S.A., Downing J.R., Cleary M.L. (2009). Hierarchical maintenance of MLL myeloid leukemia stem cells employs a transcriptional program shared with embryonic rather than adult stem cells. Cell Stem Cell.

[40] Staffas A., Arabanian L.S., Wei S.Y., Jansson A., Ståhlman S., Johansson P., Fogelstrand L., Cammenga J., Kuchenbauer F., Palmqvist L. (2017). Upregulation of Flt3 is a passive event in Hoxa9/Meis1-induced acute myeloid leukemia in mice. Oncogene.

[41] Kroon E., Krosl J., Thorsteinsdottir U., Baban S., Buchberg A.M., Sauvageau G. (1998). Hoxa9 transforms primary bone marrow cells through specific collaboration with Meis1a but not Pbx1b. Embo J..

[42] Mohr S., Doebele C., Comoglio F., Berg T., Beck J., Bohnenberger H., Alexe G., Corso J., Ströbel P., Wachter A. (2017). Hoxa9 and Meis1 Cooperatively Induce Addiction to Syk Signaling by Suppressing miR-146a in Acute Myeloid Leukemia. Cancer Cell.

[43] McIntosh B.E., Brown M.E., Duffin B.M., Maufort J.P., Vereide D.T., Slukvin I.I., Thomson J.A. (2015). Nonirradiated NOD,B6.SCID Il2rgamma-/- Kit(W41/W41) (NBSGW) mice support multilineage engraftment of human hematopoietic cells. Stem Cell Rep..

[44] Geissler E.N., McFarland E.C., Russell E.S. (1981). Analysis of pleiotropism at the dominant white-spotting (W) locus of the house mouse: A description of ten new W alleles. Genetics.

[45] Shultz L.D., Lyons B.L., Burzenski L.M., Gott B., Chen X., Chaleff S., Kotb M., Gillies S.D., King M., Mangada J. (2005). Human lymphoid and myeloid cell development in NOD/LtSz-scid IL2R gamma null mice engrafted with mobilized human hemopoietic stem cells. J. Immunol..

[46] Liu J., Zhao Z., Qiu N., Zhou Q., Wang G., Jiang H., Piao Y., Zhou Z., Tang J., Shen Y. (2021). Co-delivery of IOX1 and doxorubicin for antibody-independent cancer chemo-immunotherapy. Nat. Commun..

[47] Schiller R., Scozzafava G., Tumber A., Wickens J.R., Bush J.T., Rai G., Lejeune C., Choi H., Yeh T., Chan M.C. (2014). A cell-permeable ester derivative of the JmjC histone demethylase inhibitor IOX1. ChemMedChem.

[48] Koiwa T., Hamano-Usami A., Ishida T., Okayama A., Yamaguchi K., Kamihira S., Watanabe T. (2002). 5′-Long Terminal Repeat-Selective CpG Methylation of Latent Human T-Cell Leukemia Virus Type 1 Provirus In Vitro and In Vivo. J. Virol..

[49] Colombo A.R., Zubair A., Thiagarajan D., Nuzhdin S., Triche T.J., Ramsingh G. (2017). Suppression of Transposable Elements in Leukemic Stem Cells. Sci. Rep..

[50] Wilhelm B.T., Briau M., Austin P., Faubert A., Boucher G., Chagnon P., Hope K., Girard S., Mayotte N., Landry J.-R. (2011). RNA-seq analysis of 2 closely related leukemia clones that differ in their self-renewal capacity. Blood.

[51] Falini B., Mecucci C., Tiacci E., Alcalay M., Rosati R., Pasqualucci L., La Starza R., Diverio D., Colombo E., Santucci A. (2005). Cytoplasmic nucleophosmin in acute myelogenous leukemia with a normal karyotype. N. Engl. J. Med..

[52] Pabst C., Bergeron A., Lavallée V.-P., Yeh J., Gendron P., Norddahl G.L., Krosl J., Boivin I., Deneault E., Simard J. (2016). GPR56 identifies primary human acute myeloid leukemia cells with high repopulating potential in vivo. Blood.

[53] Donato E., Correia N., Andresen C., Karpova D., Würth R., Klein C., Sohn M., Przybylla A., Zeisberger P., Rothfelder K. (2023). Retained functional normal and preleukemic HSCs at diagnosis are associated with good prognosis in DNMT3AmutNPM1mut AMLs. Blood Adv..

[54] Lehnertz B., Pabst C., Su L., Miller M., Liu F., Yi L., Zhang R., Krosl J., Yung E., Kirschner J. (2014). The methyltransferase G9a regulates HoxA9-dependent transcription in AML. Genes Dev..

[55] Brumbaugh J., Kim I.S., Ji F., Huebner A.J., Di Stefano B., Schwarz B.A., Charlton J., Coffey A., Choi J., Walsh R.M. (2019). Inducible histone K-to-M mutations are dynamic tools to probe the physiological role of site-specific histone methylation in vitro and in vivo. Nat. Cell Biol..

[56] Assi S.A., Imperato M.R., Coleman D.J.L., Pickin A., Potluri S., Ptasinska A., Chin P.S., Blair H., Cauchy P., James S.R. (2019). Subtype-specific regulatory network rewiring in acute myeloid leukemia. Nat. Genet..

[57] Khan I., Amin M.A., Eklund E.A., Gartel A.L. (2024). Regulation of HOX gene expression in AML. Blood Cancer J..

[58] Lawrence H.J., Rozenfeld S., Cruz C., Matsukuma K., Kwong A., Kömüves L., Buchberg A., Largman C. (1999). Frequent co-expression of the HOXA9 and MEIS1 homeobox genes in human myeloid leukemias. Leukemia.

[59] Salzberg A.C., Harris-Becker A., Popova E.Y., Keasey N., Loughran T.P., Claxton D.F., Grigoryev S.A. (2017). Genome-wide mapping of histone H3K9me2 in acute myeloid leukemia reveals large chromosomal domains associated with massive gene silencing and sites of genome instability. PLoS ONE.

[60] Metzger E., Stepputtis S.S., Strietz J., Preca B.-T., Urban S., Willmann D., Allen A., Zenk F., Iovino N., Bronsert P. (2017). KDM4 Inhibition Targets Breast Cancer Stem-like Cells. Cancer Res..

