# Supplementary material for: Increasing H3K9 Methylation Level Reduces the Proliferation of Leukemic Stem Cells
Source: Cancers (Basel). 2026 Sep 14;18(18):2969. doi: 10.3390/cancers18182969 (PMC13604805; doi:10.3390/cancers18182969)

**H3K9me** OCI AML

| | CMRB 2025-12-18 10h25m51s | |
|---|---|---|
| | No. | Adj. Vol. (Int) |
| | **DMSO** | 42298.28571 |
| | **50** | 235452.2857 |
| Replica #1 | **200** | 2384429.714 |
| | **DMSO** | 16956.28571 |
| | **50** | 260839.8571 |
| Replica #2 | **200** | 1469126.571 |
| | **DMSO** | 18863.14286 |
| | **50** | 331989 |
| Replica #3 | **200** | 12192972.29 |

Image quantified (not saturated)

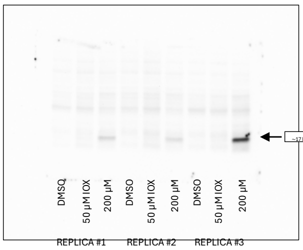


Image for viewing purposes (saturated)

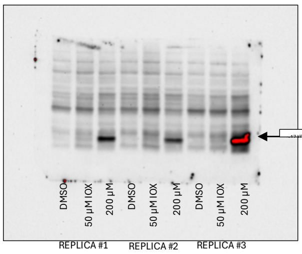


Merged colorimetric/blot

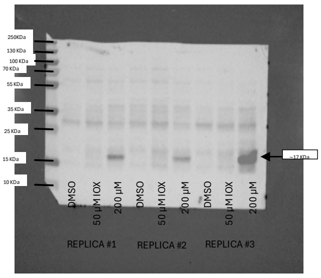


**H3K9me** OCI-AML

| | CMRB 2025-12-19 10h33m00s | |
|---|---|---|
| | | Adj. Vol. (Int) |
| | **DMSO** | 7931.2381 |
| | **50** | 2497.06557 |
| Replica #1 | **200** | 1379379.71 |
| | **DMSO** | 1239.96364 |
| | **50** | 5838.94737 |
| Replica #2 | **200** | 993905.714 |
| | **DMSO** | 9181.35345 |
| | **50** | 100698.113 |
| Replica #3 | **200** | 9305479.71 |

Image quantified (not saturated)

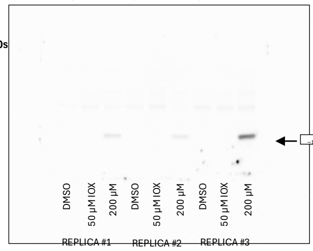


Image for viewing purposes (saturated)

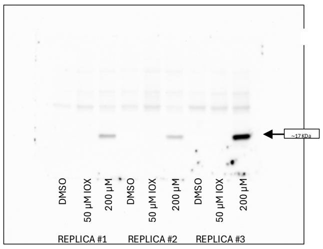


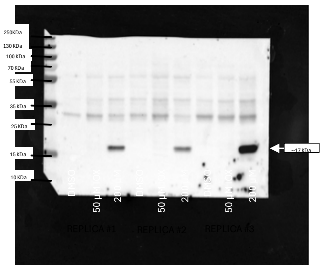


H3 TOTAL

| | CMRB 2025-12-19 14h58m05s | OCI-AML |
|---|---|---|
| | | |
| | | Adj. Vol. (Int) |
| | **DMSO** | 144370.161 |
| | **50** | 2467971.16 |
| Replica #1 | **200** | 8863407.32 |
| | **DMSO** | 453530.323 |
| | **50** | 2775128.87 |
| Replica #2 | **200** | 7785289.9 |
| | **DMSO** | 206745.742 |
| | **50** | 2189391.84 |
| Replica #3 | **200** | 7890472.39 |

Image quantified (not saturated)

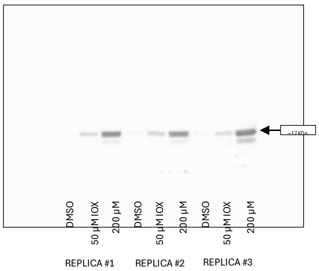


Image for viewing purposes (saturated)

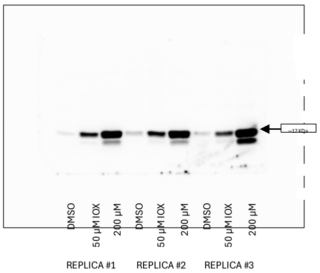


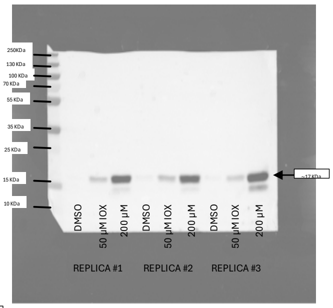


TOTAL PROTEIN TGX BLOT

| | CMRB 2025-12-17 15h06m35s | |
|---|---|---|
| | No. | Adj. Vol. (Int) |
| | **DMSO** | 306861046.68 |
| | **50** | 346558447.23 |
| Replica #1 | **200** | 402177937.58 |
| | **DMSO** | 301132246.57 |
| | **50** | 574226594.65 |
| Replica #2 | **200** | 310610629.77 |
| | **DMSO** | 343598744.83 |
| | **50** | 564386260.39 |
| Replica #3 | **200** | 847552714.32 |

Image quantified (not saturated)

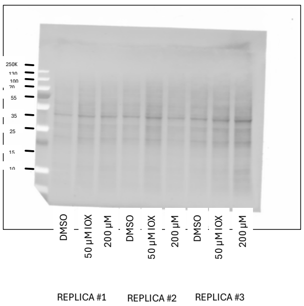


Image for viewing purposes (saturated)

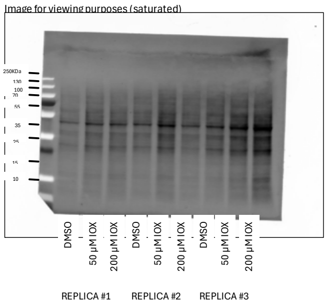

Supplement: Supplementary file 1 [file cancers-18-02969-s001.zip › FigSup4b_MDPICancers_sourceDataFile 5.pdf]
